# Sonic hedgehog: its expression in a healing cornea and its role in neovascularization

**Published:** 2009-05-18

**Authors:** Kyoko Fujita, Takeshi Miyamoto, Shizuya Saika

**Affiliations:** Department of Ophthalmology, Wakayama Medical University, Kimiidera, Wakayama, Japan

## Abstract

**Purpose:**

To examine if sonic hedgehog (Shh) is involved in tissue neovascularization by using cell culture and an animal cornea.

**Methods:**

The effects of exogenous Shh (5.0 nM), vascular endothelial growth factor (VEGF), and/or a Shh signal inhibitor (2.5 or 10.0 μM cyclopamine) on vessel-like tube formation of vascular endothelial cells were examined in vitro. The effects of Shh on the expression of angiogenic cytokines in cultured cell types were examined in cultured cells. The expression of Shh and its receptor, Patched 1 (Ptc), was examined in a vascularized mouse cornea during post-alkali burn healing. The effect of exogenous Shh on corneal neovascularization in vivo was assayed using a rat cornea system. The effect of a topical injection of cyclopamine on cauterization-induced corneal neovascularization was then studied.

**Results:**

Adding Shh promoted vessel-like tube formation of vascular endothelial cells. This effect was counteracted by addition of cyclopamine. Cyclopamine did not affect VEGF-enhanced tube formation. Shh did not affect the expression levels of angiogenic cytokines in cultured cell types. mRNA and protein expression levels of Shh and Ptc were under the detection limit in an uninjured cornea, but Shh but not Ptc was upregulated in a healing, alkali-burned, vascularized cornea. Exogenous Shh promoted neovascularization (NV) formation in vivo in a rat cornea. Topical cyclopmine blocked Gli signaling (blocked translocation of Gli3) and the length of neovascularization in the peripheral cornea post-cauterization as compared with the control vehicle-treated cornea.

**Conclusions:**

Shh enhances endothelial tube formation independently through VEGF signaling in vitro. Shh signaling is involved in the development of unfavorable corneal neovascularization in animal corneas.

## Introduction

Vascular growth (neovascularization, NV) might be beneficial to tissue repair in an injured tissue. For example, vessel growth is essential in a survival skin graft [1]. However, the cornea is an avascular tissue of the eye and must remain transparent to refract light properly. Therefore, injury-induced NV is one of the unfavorable factors that potentially impairs patients’ vision. Cytokines/growth factors are believed to orchestrate cell behavior during the development of NV in a healing cornea. Although transforming growth factor (TGF) β, vascular endothelial cell growth factor (VEGF), and tumor necrosis factor (TNF) α are believed to have central roles in corneal NV development, other factors must be involved [2,3]. Sonic hedgehog (Shh) is a morphogen and is a secretory protein of molecular weight 20 kDa. Shh is known to be involved not only in embryonic organogenesis but also in development of malignant neoplasms such as basal cell carcinoma or other cancers [4-12]. Upon Shh binding to the receptor composed of patched 1 (Ptc) and Smoothend (Smo), the Shh signal transmitter, Gli members, are activated and translocated to the cell nuclei for gene expression regulation. For example, Shh signaling upregulates cyclin D1, leading to cell proliferation promotion [13].

As for eye tissues, the pathobiology of Shh signaling is to be investigated. We previously reported that Shh but not the Ptc Shh receptor is upregulated in the healing rat corneal epithelium post-debridement and that Shh promotes cell proliferation in the healing epithelium of a mouse cornea [14]. It has also been reported that Shh signaling is beneficial in wound healing in the skin and heart, but the effects might depend on the induction of neovascularization, which is critical in the repair process in such tissues [15,16]. Moreover, Shh is reportedly involved in not only cell proliferation regulation but also NV development during the progression of malignant tumors [17,18].

However, the role of Shh in a complicated corneal healing process such as that seen in a cornea burned with alkali has not been investigated. In such, cornea NV could be one of the key factors that influences unfavorable stromal opacification in a healing cornea, such as the generation of myofibroblasts.

In the present study, we first investigated the role of Shh signaling in NV formation by using a co-culture system of vein endothelial cells and fibroblasts. We then examined if Shh is expressed in a post-alkali burned, neovascularized, healing corneal stroma and if exogenous Shh promotes NV formation in a rat cornea. We finally examined if blocking Shh signaling suppresses NV in a cornea by using cyclopamine. This compound binds Ptc and Smo in the Shh receptor complex and inhibits Shh signaling [14,19].

## Methods

Experiments were approved by the Animal Care and Use Committee of Wakayama Medical University (Wakayama, Japan) and conducted in accordance with the Association for Research in Vision and Ophthalmology Statement for the Use of Animals in Ophthalmic and Vision Research.

### Assay for vessel-like tube formation by vascular endothelial cells

A commercially available co-culture system of human umbilical vein endothelial cells (HUVECs) and human fibroblasts (NV kit; Kurabo, Tokyo, Japan) was used according to the manufacturer’s protocol. In this kit, HUVECs form vessel-like tube tissues that are labeled with anti-CD31 antibody on a fibroblast feeder layer. HUVECs were seeded on a fibroblast feeder layer in the wells of two-well culture plates. Recombinant Shh (5.0 nM; R&D systems, Minneapolis, MN) alone or Shh (5.0 nM) and cyclopamine (2.5 or 10.0 μM; Toronto Research Chemicals, North York, Ontario, Canada) was added to the culture medium at days 1, 2, 4, and 7 of culture. The control culture did not contain these compounds. The cultures were fixed with 70% ethanol on day 11 of culture and processed for immunocytochemistry for CD31. The antibody complex was visualized with diaminobenzidine as previously reported [14]. Four wells were prepared for each culture condition. The total length and the number of branching points were counted in five independent points in each well.

To examine if Shh signaling is involved in VEGF-dependent vessel-like tube formation, we conducted the following experiment. The culture was treated with recombinant VEGF (10.0 ng/ml) or VEGF plus cyclopamine (2.5 or 10.0 μM) and assessment of tube formation was as described above. Statistical analysis was conducted by employing Turkey-Kramer’s test, and p<0.05 was taken as statistically significant.

### Alkali burn model in adult mice

An alkali burn was produced with 3 ml of 1 N topical NaOH in the cornea of an adult C57BL/6 mouse (n=42) under general and topical anesthesia as previously reported [20,21] and allowed to heal for up to 5, 10, or 20 days. Cryosections and paraffin sections fixed with cold acetone were processed for immunohistochemistry with goat anti-Shh antibody or anti-Ptc antibody (20 μg/ml in phosphate-buffered saline [PBS]; R&D systems). Histology was examined by using hematoxylin and eosin (HE) staining.

Real-time reverse transcription polymerase chain reaction (RT–PCR) was also performed to detect mRNAs of *Shh* and *Ptc* in a healing, alkali-burned cornea. The cornea of a C57BL/6 mouse (n=29) was burned with 1 N NaOH as described above. An uninjured cornea served as the control. Two days later total RNA was extracted from the excised cornea. The RNA sample was analyzed using TaqMan real-time RT–PCR, (Applied Biosystems, Tokyo, Japan) a primer set, and a TaqMan probe for *Shh* or *Ptc* (ABI Assay ID: Mm00436527 or Mm00436031)

### Effects of exogenous Shh on expression of angiogenic cytokine expression in cultured cells

To examine if Shh is capable of inducing angiogenic cytokines in epithelial and mesenchymal cell types, we performed an in vitro experiment and an enzyme-linked immunosorbent assay (ELISA). The Araki-Sasaki human corneal epithelial cell line or mouse fibroblasts were cultured until confluency in either Dulbecco’s modified Eagle’s medium (DMEM) supplemented with 5% fetal calf serum or DMEM supplemented with 15% fetal calf serum. Each cell type was then incubated in serum-free DMEM for 24 h. The cells were then cultured in the presence of recombinant Shh (2.5 nM, 5.0 nM, 10.0 nM; R&D systems). After an incubation for 24 h, the culture medium was harvested and processed for ELISA assay (Quantikine; R&D Systems) of TGFβ1, VEGF, or Monocyte Chemotactic Protein-1 (MCP-1). Cells were also processed for total RNA extraction and real-time RT–PCR for mRNAs of human or mouse *TGFβ1*, *VEGF*, and *MCP-1* using the TaqMan one step system with the primers and TaqMan probes as previously reported [2,22,23].

### Effect of cyclopamine on development of corneal neovascularization in vivo

Corneal NV from the limbal vessels was induced by two methods, central corneal cauterization in one eye of individual mice using implantation of a Shh-containing polymer pellet to the stroma or cauterization by a disposable Optemp tool (Mod-Tronic Instruments, Brampton, ON, Canada) as previously reported [3].

We first examined the direct effect of exogenous Shh on corneal NV formation using implantation of a Shh-containing polymer pellet to the corneal stroma. Adult Wistar rats (n=20) were used. The polymer pellets were prepared as follows. Polyethylene-co-vinyl acetate (Sigma-Aldrich, St. Louis, MO) was dissolved in 1,1-dinitroethane (30% solution in ethylene chloride) in a water bath (37 °C) for 30 min at the final concentration of 100 mg/ml. The polymer solution was set in a glass dish and dissected to obtain small pieces of the pellets (1 mm×1 mm×0.1 mm). Four treatment groups were prepared: 1) control without exogenous agents; 2) a polymer pellet containing recombinant Shh (50 μg/ml in phosphate-buffered saline [PBS]; R&D systems); 3) a polymer pellet with cyclopamine (2.5 mg/ml in PBS, Toronto Reserch Chemicals); and 4) with recombinant Shh (50 μg/ml in PBS + cyclopamine [2.5 mg/ml in PBS]). Each pellet was implanted into the peripheral corneal stroma as follows. A pocket was produced with a surgical blade in the peripheral cornea of a Wistar rat (n=20) that was generally anesthetized with intra peritoneal (i.p.) entobarbital sodium. Each pellet was inserted into the pocket of the stroma, and ofloxacin ointment was topically applied to reduce the risk of bacterial infection. Ten days after, We evaluated development of corneal NV. The length of the NV was statistically analyzed by using the Turkey-Kramer method, and a p<0.05 was taken as statistically significant.

Next, to know the role(s) of endogenous Shh on cauterization-induced NV formation, cyclopamine (2.5 mg/ml in PBS) or PBS alone was injected into retrobulbar tissue using a hypodermic needle at the time of cauterization. Experimental mice were sacrificed seven days after injury. The eye was then enucleated and subjected to HE histology and cryosection for immunohistochemistry of CD31 and nuclear translocation of Gli3, the signaling molecule of Shh. The length of NV in the peripheral cornea was measured and analyzed using the Wilcoxon signed ranks test. P values less than 0.05 were taken as statistically significant.

## Results

### Effects of exogenous Shh on expression of angiogenic cytokine expression in cultured cells

**Figure 1:**
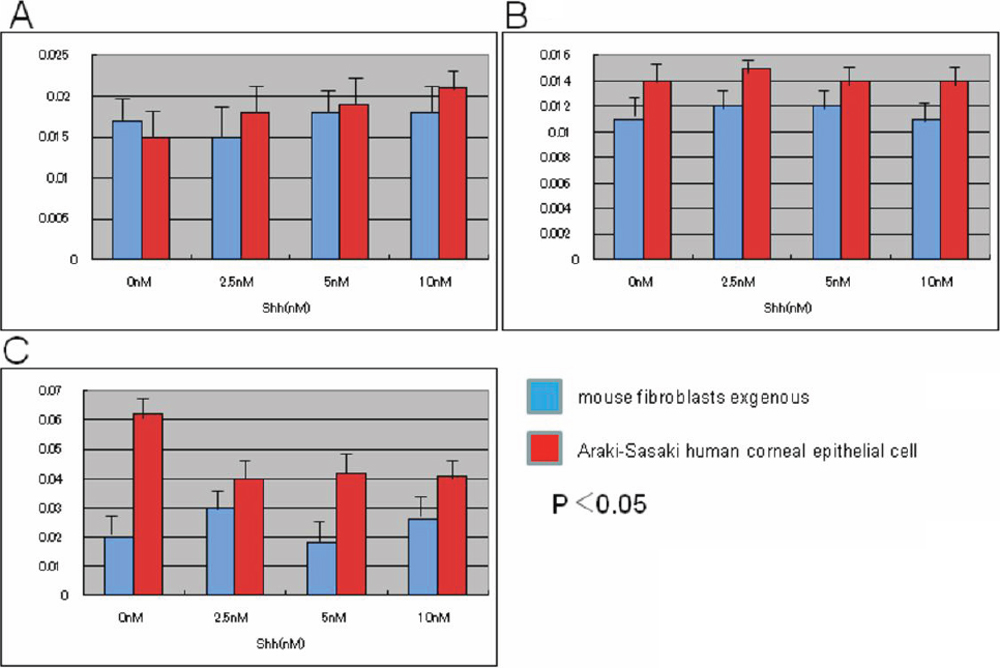
Effects of exogenous Sonic hedgehog on the expression of angiogenic cytokine expression in cultures of mouse fibroblasts and Araki-Sasaki corneal epithelial cells. In both cell types, adding exogenous Shh does not affect mRNA expression levels of transforming growth factor β1 (*TGFβ1*; **A**), monocyte/macrophage-chemoattractant protein-1 (*MCP-1*; **B**), or vascular endothelial growth factor (*VEGF*; **C**).

In both cell types of Araki-Sasaki corneal epithelial cells and mouse fibroblasts, exogenous Shh did not affect the protein and mRNA expression level of TGFβ1, VEGF, and MCP-1 until 24 h after Shh (2.5 μm, 5.0 μm, 10.0 μm) incubation (Figure 1).

### Vessel-like tube tissue formation by HUVECs

**Figure 2:**
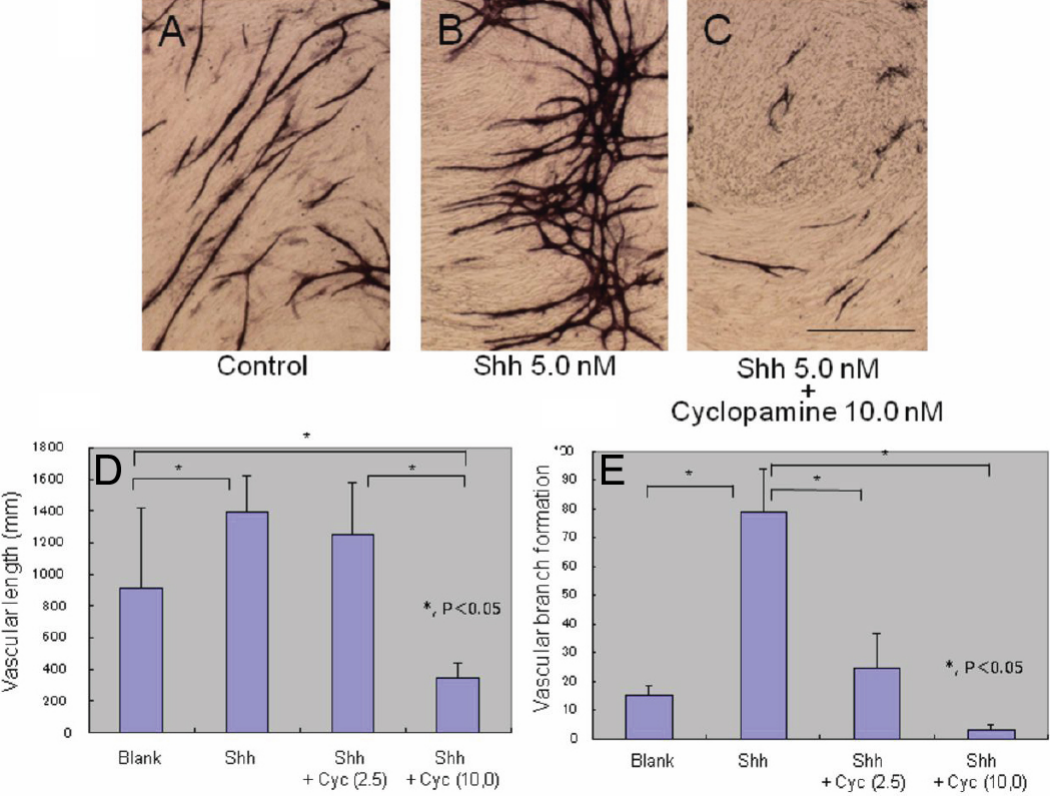
Effects of exogenous Sonic hedgehog on in vitro angiogenesis in HUVECs on fibroblast feeder layer. Exogenenous Sonic hedgehog (Shh) enhances CD31-labeled, vessel-like tube tissue formation when compared with the control co-culture. Such enhancement of tube formation by exogenous Shh is suppressed by further addition of cyclopamine (**A**-**C**). Bar, 50 mm. Adding recombinant Shh at 5.0 nM promoted the formation of vessel-like tube formation in HUVECs as evaluated by the total length of the structure (**D**) and the number of the branching points (**E**). Further addition of cyclopamine (2.5 or 10.0 μM) suppressed the elongation and branching of the tube structure.

When evaluated by the measurement of the total length of vessel-like tubes formed by HUVECs, data in Shh (5.0 nmol/l) treated cultures and in Shh (5.0 nmol/l) plus cyclopamine (2.5 mmol/l) treated cultures were significantly higher than the data in the control culture. Further addition of cyclopamine at 2.5 mM and 10.0 mM significantly suppressed Shh-induced vessel-like tube formation as evaluated by its total length (Figure 2). Similarly, adding Shh enhanced the total number of branching points of the tube and such enhancement was counteracted by the further addition of cyclopamine.

**Figure 3:**
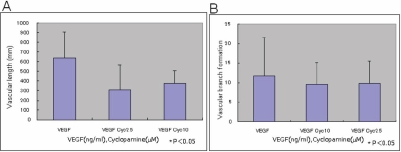
Effects of cyclopamine on in vitro angiogenesis in HUVECs on fibroblast feeder layer. Tube formation in the presence of vascular endothelial growth factor (VEGF) was not affected by the addition of cyclopamine at the concentrations of 2.5 and 10.0 ng/ml as revealed by the evaluation of the total length of CD31-positive tissue (**A**) and the numbers of its branching (**B**).

The effect of cyclopamine on VEGF-supported vessel-like tube formation by HUVECs was also examined. The addition of cyclopamine (2.5 mM and 10.0 mM) to VEGF treated cultures did not significantly alter the total length and the number of branching points in vessel-like tube structure (Figure 3).

### Histology of an alkali-burned cornea

**Figure 4:**
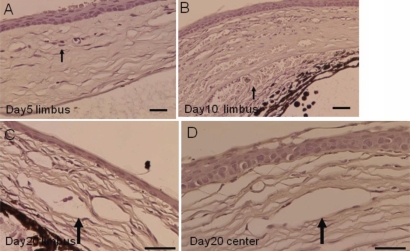
Histology of burned corneas stained with hematoxylin and eosin. On day 5 (**A**) and 10 (**B**), neovascularization is observed in the limbal region of an alkali-burned cornea. Neovascularization is detected even in the central cornea at day 20 (**C**,**D**). Bar, 50 μm.

At day 5 post-alkali burn, minor NV was observed in the peripheral cornea adjacent to the limbus. At day 20, marked NV was found to grow from the limbus toward the central cornea (Figure 4).

### *Shh* mRNA expression in an alkali-burned cornea

Real-time RT–PCR was performed to detect Shh mRNA expression in a healing, alkali-burned cornea. The expression level of *Shh* mRNA was under the detection limit in the uninjured cornea while *Shh* mRNA expression was certainly detected at a markedly high level (approximately 227 fold compared with control) in an alkali-burned cornea. On the other hand, *Ptc* mRNA expression level was unchanged during wound healing in an alkali-burned cornea (data not shown).

### Immunohistochemistry

**Figure 5:**
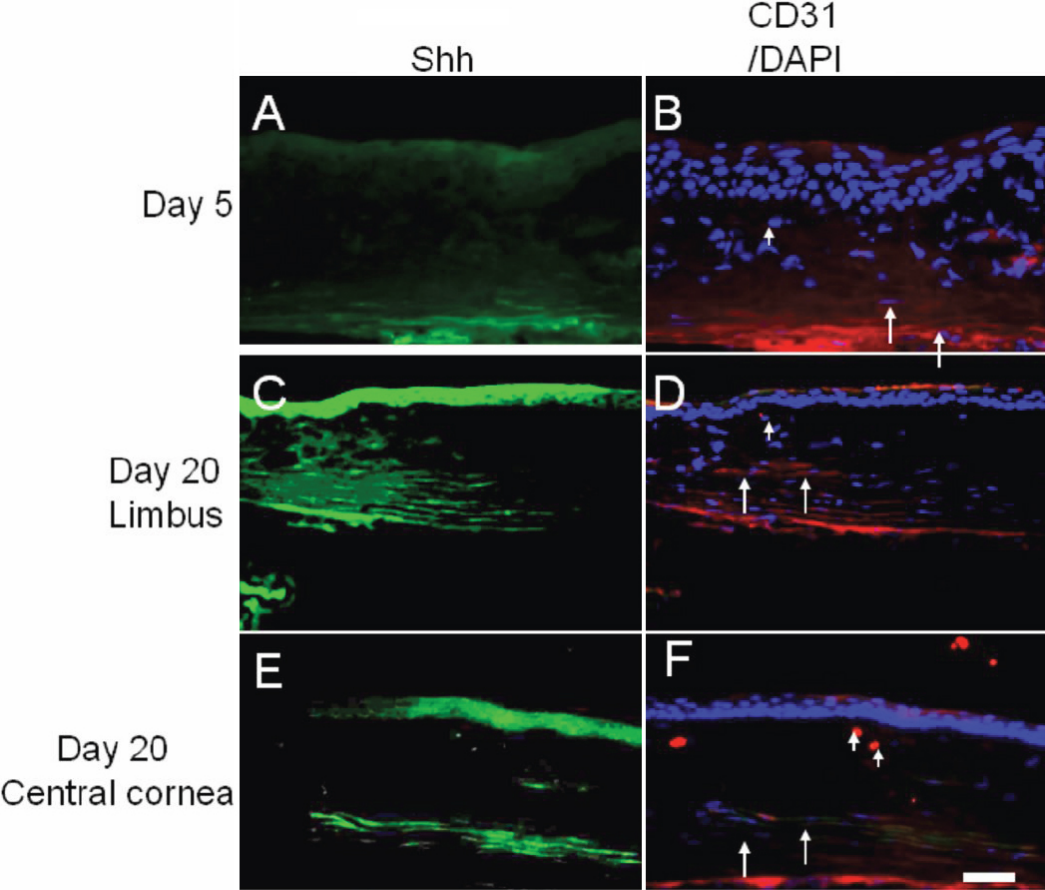
Expression pattern of Sonic hedgehog and CD31 in alkali-burned corneas. On day 5 (**A**,**B**) and 20 (**C**,**D**), neovascularization in the limbal region was stained for Sonic hedgehog (Shh; FITC). The healing epithelium as well as the stroma were labeled for Shh (**C**). On day 20, CD31-labeled (Rhodamin) neovascularization was observed in the area of the corneal stroma stained for Shh (**E**,**F**). Bar, 50 μm.

To define which cell type(s) express(es) Shh, we then performed immunohistochemistry. As previously reported by us [14], Shh was not detected in the corneal stroma of an uninjured cornea (data not shown) but was upregulated in both the healing epithelium and stroma (Figure 5A,C,E) until day 20. Corneal neovascularization as labeled for CD31 was observed in the cornea from day 5 to day 20 post-burn (Figure 5B,D,F). Shh expression was more marked in the area of stroma with neovascularization.

### Role of exogenous Shh and cyclopamine on development of corneal stromal neovascularization in vivo

**Figure 6:**
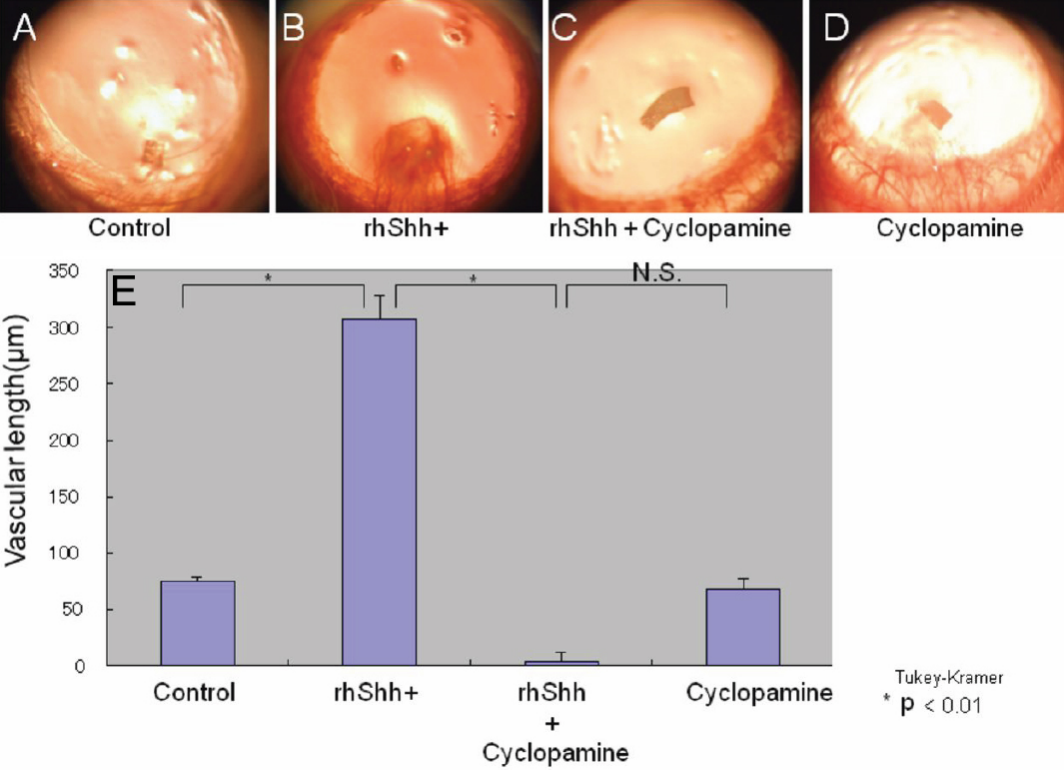
Effect of exogenous Sonic hedgehog on the development of corneal neovascularization in vivo. **A**-**D**: Implantation of a polymer pellet-containing recombinant Shh induced marked NV in the stroma from limbal vessels (**B**) as compared with control corneas (**A**), indicating that Shh has a NV-promoting effect in vivo like in vitro. Pellet containing cyclopamine reduced NV formation as compared with control (**C**, **D**), indicating that endogenous Shh also has a promoting role in the development of corneal NV. Cyclopamine indeed counteracted exogenous Shh-induced NV. **B** shows the length of the NV developed from limbus in each group of treatment (**E**).

We first examined a direct effect of exogenous Shh on corneal NV formation. We employed implantation of a Shh-containing polymer pellet to the corneal stroma. Implantation of a polymer pellet containing recombinant Shh induced marked NV in the stroma from the limbal vessels compared with control corneas, indicating that Shh has a NV-promoting effect in vivo just as it does in vitro. A pellet containing cyclopamine reduced NV formation compared with the control, indicating that endogenous Shh also has a promoting role in the development of corneal NV. Cyclopamine indeed counteracted exogenous Shh-induced NV. (Figure 6)

### Effect of cyclopamine on the development of cauterization-induced corneal neovascularization in vivo

**Figure 7:**
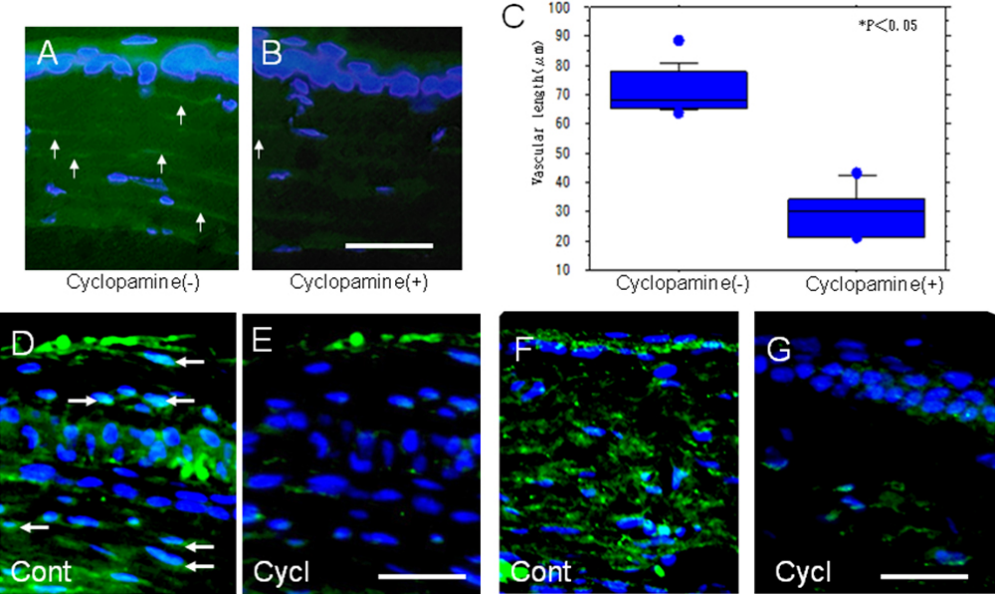
Effect of cyclopamine on the development of corneal neovascularization in vivo. **A**, **B**: Cauterization in the central cornea induced neovascularization (NV) in the peripheral cornea (arrows) in the control group. Such NV formation is seen to be suppressed in the cyclopamine-treated group (**B**) when compared with the control (**A**). **C**: The length of NV in the peripheral cornea from the limbus is shown. The NV length is significantly reduced in the cyclopamine group as compared with the control (p<0.05). **D**, **E**: Nuclear translocation of Gli3 (arrows) is detected in the corneal epithelium and stromal cells of the peripheral cornea in PBS-treated control group at day 7 (**D**) while it is not observed in a cornea treated with topical cyclopamine (**E**). **F**, **G**: Induction of Sonic hedgehog is also seen to be suppressed in a treatment group (**F**) when compared with the control group (**G**). Nuclear counterstaining was done with DAPI. Bar, 50 μm.

We then examined the role(s) of endogenous Shh on injury-induced corneal NV formation, although the experiment above by using an implantation of a pellet to the cornea suggested both exogenous and endogenous Shh is involved in NV development. Cauterization in the central cornea induced NV in the peripheral cornea as previously reported [20,21]. Topical cyclopamine decreased the length of neovascularization in the peripheral cornea post-cauterization when compared with the control vehicle-treated cornea seven days after injury (Figure 7A-C). In these specimens, we then examined the expression of Shh and the nuclear translocation of Gli3. Immunohistochemistry showed that not only the nuclear translocation but also further induction of Shh in tissue were suppressed by topical treatment with cyclopamine (Figure 7D-G).

## Discussion

In the present study, we first examined if exogenous Shh promotes NV formation. We then conducted an in vitro co-culture experiment by using fibroblast feeder and HUVECs. This in vitro experiment showed that adding recombinant Shh accelerated vessel-like tube formation by HUVECs, suggesting a promoting role of Shh in NV development. Counteraction of cyclopamine to Shh-enhanced tube formation further suggested the role of Shh there. The total length of tube-like tissue might depend on endogenous Shh because its total length in cyclopamine culture (data not shown) or Shh+cyclopamine culture (Figure 2B) was significantly less compared with that seen in the control culture. It was reported that Shh upregulates VEGF in endothelial progenitor cells isolated from human peripheral blood but did not upregulate VEGF in HUVECs, primary dermal microvascular endothelial cells, or fibroblasts [24,25]. Cyclopamine did not affect the total length or the number of branching points of the in vitro vessel-like structure in the presence of exogenous VEGF, suggesting that Shh signaling is independent of VEGF signaling. Exogenous Shh promoted the development of corneal NV. We then tested whether this phenomenon is observed in vivo in animal corneas. We detected upregulation of Shh in a healing stroma of an alkali-burned neovascularized mouse cornea, suggesting that Shh might have a role in the development of NV in a healing cornea. The expression level of Ptc, the Shh receptor, did not change during healing in an alkali-burned cornea. As discussed later, blocking endogenous Shh by cyclopamine attenuated NV formation in a mouse cornea. Shh reportedly enhances NV-dependent tissue repair in skin and heart [15,16]. However, NV is unfavorable to maintain/recover the vision/transparency of a patient with a corneal injury and thus should be suppressed.

As described above in the in vitro experiment, the VEGF-promoted tube formation was not affected by further addition of cyclopamine, suggesting that Shh signaling is not not located downstream of VEGF signaling. It has been reported that cyclopamine attenuates vasculogenesis in the developing mouse heart in association with suppressing VEGF expression [26]. It has also been reported that Shh upregulates the expressions of VEGF and angiopoietin-1,-2 in cultured human lung fibroblasts [27]. On the other hand, exogenous Shh does not affect the expression level of VEGF in mouse vascular endothelial cells in vitro [28]. Explanations for this discrepancy include the differences in condition, differences in cell types, and in vivo versus in vitro. In vivo Shh signaling might affect VEGF expression in the cells adjacent to the new vessels, i.e., fibroblasts or bone marrow-derived pericytes [29]. During the in vitro condition, the newly developed vessel-like structure is composed of vascular endothelial cells. The in vivo condition behavior of such pericytes or other vessel supporting mesenchymal cells might be modulated by Shh signaling.

These results finally led us to hypothesize that blocking Shh signaling might have a beneficial inhibitory effect on NV development in the cornea. To explore this hypothesis we conducted an in vivo experiment. Shh, but not the Ptc receptor, was upregulated in a vascularized healing mouse cornea post-alkali burn. The exact mechanism of upregulation of Shh expression in a healing, injured cornea needs to be explored. We have reported that the healing corneal epithelium following debridement express Shh [14], suggesting that activation of cells upon injury might switch on its expression. We therefore examined a direct effect of exogenous Shh on corneal NV formation. We employed an implantation of a Shh-containing polymer pellet to the corneal stroma. Implantation of a polymer pellet containing recombinant Shh induced marked NV in the stroma from limbal vessels when compared with control corneas, indicating that Shh has a NV-promoting effect in vivo, the same as in vitro. A pellet-containing cyclopamine reduced NV formation when compared the with control, indicating that endogenous Shh also has a promoting role in the development of corneal NV. Cyclopamine indeed counteracted exogenous Shh-induced NV. We then examined the roles of endogenous Shh on injury-induced corneal NV formation, although the experiment above suggested that both exogenous and endogenous Shh is involved in NV development. Cauterization at the center of the cornea induced NV development in the corneal periphery, and this NV formation was suppressed by topical administration (injection into retrobulbar tissue) of cyclopamine, indicating that cyclopamine’s inhibitory effect on NV formation was even observed in vivo. In the present study, we used injections into the retrobulbar tissue instead of systemic administration. Although animal species are different (a primate versus mouse), it was reported that the concentration of an anti-cancer drug in the vitreous humor was much higher when topically administered to the orbit as compared with via systemic administration [30]. These results indicate that endogenous Shh/Gli signaling is actually involved in the development of corneal NV.

Shh signaling is also reportedly involved in NV formation in experimental models of choroidal NV or retinal NV. This report also shows that Shh activation is located upstream of VEGF in experimental retinal neovascularization under retinal hypoxic conditions because inhibition of the Shh pathway results in the reduction of VEGF level along with that of Patched-1 (Ptch1), a canonical Shh target. In the present study of a mouse cornea, blocking Shh signaling might directly affect the cells involved in NV tissue because blocking Shh signaling by using cyclopamine does not affect the expression level of VEGF in cultured corneal epithelial cells and fibroblasts. The exact mechanism underlying this discrepancy is has yet to be explored [31].

Our experiment suggests that Shh signaling is a potential target to prevent and/or treat unfavorable NV formation in the cornea.
